# Response of wild bee diversity, abundance, and functional traits to vineyard inter‐row management intensity and landscape diversity across Europe

**DOI:** 10.1002/ece3.5039

**Published:** 2019-03-12

**Authors:** Sophie Kratschmer, Bärbel Pachinger, Martina Schwantzer, Daniel Paredes, Gema Guzmán, José A. Goméz, José A. Entrenas, Muriel Guernion, Françoise Burel, Annegret Nicolai, Albin Fertil, Daniela Popescu, Laura Macavei, Adela Hoble, Claudiu Bunea, Monika Kriechbaum, Johann G. Zaller, Silvia Winter

**Affiliations:** ^1^ Institute for Integrative Nature Conservation Research University of Natural Resources and Life Sciences Vienna Austria; ^2^ Estación Experimental de Zaidín CSIC Granada Spain; ^3^ Institute for Sustainable Agriculture CSIC Córdoba Spain; ^4^ UMR 6553 EcoBio University Rennes 1, Biological Station of Paimpont Paimpont France; ^5^ UMR 6553 EcoBio University Rennes, CNRS Rennes France; ^6^ Research Department SC JIDVEI SRL Jidvei Romania; ^7^ UNIMORE, University of Modena and Reggio Emilia Italy; ^8^ University of Agriculture Science and Veterinary Medicine Cluj Napoca Romania; ^9^ Institute of Zoology University of Natural Resources and Life Sciences Vienna Austria; ^10^ Division of Plant Protection University of Natural Resources and Life Sciences Vienna Austria

**Keywords:** Apiformes, ecosystem services, floral resource availability, functional traits, GLMM, Shannon Landscape Diversity Index, vegetation management, viticulture landscapes

## Abstract

Agricultural intensification is a major driver of wild bee decline. Vineyards may be inhabited by plant and animal species, especially when the inter‐row space is vegetated with spontaneous vegetation or cover crops. Wild bees depend on floral resources and suitable nesting sites which may be found in vineyard inter‐rows or in viticultural landscapes. Inter‐row vegetation is managed by mulching, tillage, and/or herbicide application and results in habitat degradation when applied intensively. Here, we hypothesize that lower vegetation management intensities, higher floral resources, and landscape diversity affect wild bee diversity and abundance dependent on their functional traits. We sampled wild bees semi‐quantitatively in 63 vineyards representing different vegetation management intensities across Europe in 2016. A proxy for floral resource availability was based on visual flower cover estimations. Management intensity was assessed by vegetation cover (%) twice a year per vineyard. The Shannon Landscape Diversity Index was used as a proxy for landscape diversity within a 750 m radius around each vineyard center point. Wild bee communities were clustered by country. At the country level, between 20 and 64 wild bee species were identified. Increased floral resource availability and extensive vegetation management both affected wild bee diversity and abundance in vineyards strongly positively. Increased landscape diversity had a small positive effect on wild bee diversity but compensated for the negative effect of low floral resource availability by increasing eusocial bee abundance. We conclude that wild bee diversity and abundance in vineyards is efficiently promoted by increasing floral resources and reducing vegetation management frequency. High landscape diversity further compensates for low floral resources in vineyards and increases pollinating insect abundance in viticulture landscapes.

## 1 INTRODUCTION

Wild bees and honey bees are important pollinators of crops (Brittain, Williams, Kremen, & Klein, 2013; Klein et al., 2007) and wild plants (Fontaine, Dajoz, Meriguet, & Loreau, 2006). Pollination efficiency of different crops is strongly related to wild bee species diversity (Földesi et al., 2015; Winfree et al., 2018) as well as functional diversity (Fontaine et al., 2006; Garibaldi et al., 2015). Research demonstrated that wild bees are threatened by intensive agricultural practices (Kremen, Williams, & Thorp, 2002) such as high pesticide application (Woodcock et al., 2017), and/or frequent soil tillage (Williams et al., 2010), which result in reduction of floral resource availability (Williams et al., 2015) and contribute to landscape simplification (Senapathi, Goddard, Kunin, & Baldock, 2017).

Wild bee diversity, abundance, and pollination are strongly positively affected by the enhanced quantity and quality of floral resources (Williams et al., 2015), increased landscape heterogeneity (Andersson, Birkhofer, Rundlöf, & Smith, 2013), and the proportion of (semi‐) natural areas in agricultural landscapes (Nicholson, Koh, Richardson, Beauchemin, & Ricketts, 2017). However, wild bee species composition is differently affected by environmental disturbances and landscape configuration (Carrié et al., 2017; Hopfenmüller, Steffan‐Dewenter, & Holzschuh, 2014) because functional traits are closely related to habitat requirements (Williams et al., 2010).

Vineyards cover about 7.6 million hectares worldwide (OIV, 2018). The commercial grape vine (*Vitis vinifera* L.) is self‐pollinated and wind pollinated, thus pollination by insects only plays a minor role for grape yield (Cabello Saenz, Luis Villota, & Tortosa Tortola, 1994). Bees were rarely observed foraging on grapevine flowers (Vorwohl, 1977), but vineyards can provide habitats for wild bees to increase pollination for insect‐pollinated crops, fruit trees, cover crops, and wild plants. Maintaining wild bee diversity is essential for the resilience of pollination services (Bartomeus et al., 2013; Brittain, Kremen, & Klein, 2013) and also enhances diversity of associated plants pollinated by wild bees (Biesmeijer et al., 2006). Improving habitats for pollinators simultaneously enhances ecosystem services like biological pest control, soil and water quality protection, or landscape aesthetics (Wratten, Gillespie, Decourtye, Mader, & Desneux, 2012). Establishing and maintaining noncrop flowering areas within the farmland matrix promotes the native plant community, provides habitats for a range of insects, bird and mammals, and thus contributes to biodiversity conservation (Wratten et al., 2012). Further, a spill‐over effect of flower visitation rates in insect‐pollinated crops from field margins was observed for wild bees, which increased crop yields in closer proximity to field margins (Woodcock et al., 2016). As winegrowers experience an increased consumer demand for eco‐friendly produced wine (Schütte & Bergmann, 2019), establishing flower‐rich habitats for wild bees in vineyards can be used for marketing.

Depending on the vegetation management intensity, vineyard inter‐rows are comparable with field margins or wildflower strips in agricultural landscapes, which increase wild bee diversity (Haaland, Naisbit, & Bersier, 2011). Winegrowers manage inter‐row vegetation by tillage, mulching, or herbicide application to mitigate potential water and/or nutrient competition between the vines and the inter‐row vegetation (Pardini, Faiello, Longhi, Mancuso, & Snowball, 2002). The intensity of this disturbance varies among wine‐growing areas across Europe according to local pedological and climatic conditions.

Wild bees in vineyards have been shown to benefit from biodiversity‐friendly management practices and from mosaics of semi‐natural elements within the viticultural landscape (Kehinde & Samways, 2014a, 2014b; Kratschmer et al., 2018). Further, species characterized by certain traits may respond similarly to a certain vegetation management measure or landscape configuration in wine‐growing areas. For example, ground‐nesting species could benefit from undisturbed soil conditions for nesting in permanently vegetated inter‐rows. Further, larger species may compensate low landscape diversity with their increased activity range and forage in more fragmented landscapes (Zurbuchen et al., 2010). A meta‐analysis included only two studies about the effects of vineyard vegetation management on pollinators and concludes that knowledge about the effects of inter‐row vegetation management on wild bee diversity is scarce (Winter et al., 2018). Further until now, studies about wild bee diversity and functional traits in response to vineyard management and in relation to landscape diversity in different climatic regions (i.e., different European countries) have not yet been carried out.

We hypothesized that vegetation management intensity, floral resource availability, and the surrounding landscape diversity affect wild bee diversity, abundance, and functional traits in vineyard inter‐rows across Europe. We expected that inter‐row vegetation management effects on bees would be less pronounced in vineyard with higher floral resource availability and in heterogeneous than in simpler landscapes.

## 2 MATERIALS AND METHODS

### 2.1 Study sites

**Figure 1:**
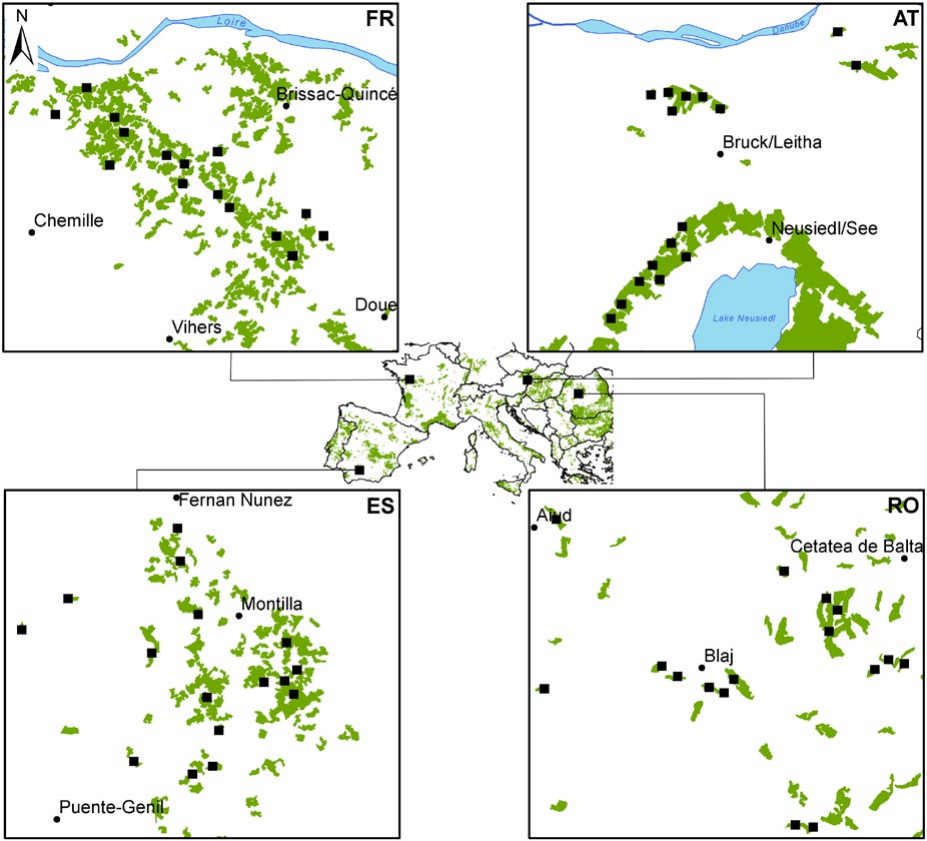
Maps of studied wine‐growing areas across Europe. FR: Loire Valley, AT: Carnuntum and Neusiedler See‐Hügelland, RO: Târnave and ES: Montilla Moriles. Green shading: Viticulture areas according to CORINE land cover (EEA, 2017). Squares: Location of studied vineyards and wine‐growing areas

This study was conducted in four viticultural areas across Europe (Spain, France, Austria, and Romania) in 2016. The locations of the viticultural areas (Figure 1) cover three European climate zones: warm Mediterranean climate in southern Spain (Montilla Moriles in Andalusia; 37°35′N, 4°38′W), temperate oceanic climate in North‐Western France (Coteaux‐du‐Layon in Loire Valley; 47°23′N, 0°42′E), and temperate continental climate in Eastern Austria (Carnuntum; 48°6′N, 16°51′E and Neusiedler See‐Hügelland; 47°52′N, 16°37′E Lower Austria and Burgenland) and Central Romania (Târnave in Transylvania; 46°13′N, 24°06′E).

In total, 63 vineyards were investigated that ranged in age from 5 to 61 years. The distance between the vines (in‐row) ranged from 0.75 to 1.9 m, and the inter‐row width varied between 1.5 and 3 m. Three different intensities of inter‐row vegetation management were studied (Table 1): (a) permanent vegetation cover without any disturbance for at least 5 years (Austria, France, and Romania), (b) temporary vegetation cover in every second inter‐row (Austria and Romania) or in every inter‐row during the winter season (Spain) by tillage, and (c) bare soil management through frequent soil tillage (Spain and Romania) and/or application of herbicides (Spain, France) in all inter‐rows. Tillage depths ranged between 5 and 40 cm across the countries. In each inter‐row, the vegetation coverage (%) was estimated twice a year (at the beginning of the vegetation period and 2 months later) in four 1 × 1 m subplots. The averaged vegetation cover per inter‐row differed significantly (Kruskal–Wallis test: *χ* ^2 ^= 38.50; *df *= 2; *p* ≤ 0.001) among the management intensities. The Spanish inter‐rows with temporary vegetation cover were managed more intensively compared to the temporary vegetated inter‐rows in Austria and Romania which resulted in a comparatively lower vegetation cover (Table 1). Mulching was done 1–5 times in permanently and temporary vegetated inter‐rows. All studied vineyards—with the exception of seven Spanish vineyards with deficit drip irrigation—were rainfed.

**Table 1:** Mean ± *SD* vegetation coverage (%) per vegetation management intensity, method of vegetation management, and number of management events per year in the studied countries

Country	Vegetation coverage (%) and no. of vineyards			Vegetation management		Landscape circles	SHDI
	Permanently vegetation	Temporary vegetation	Bare soil	Method	Events per year		
AT	82.73 ± 11.5	82.93 ± 14.5		Tillage	1−3	16	1.56 ± 0.3
	*n* = 7	*n* = 9	*n* = 0				
ES		56.08 ± 23.8	19.95 ± 19.6	Tillage and/or Herbicides	1−4	16	1.26 ± 0.2
	*n* = 0	*n* = 8	*n* = 8				
FR	96.37 ± 2.9		21.08 ± 19.6	Herbicides	1−4	15	1.54 ± 0.2
	*n* = 8	*n* = 0	*n* = 7				
RO	63.56 ± 13.7	63.45 ± 15.6	35.06 ± 12.1	Tillage	2−5	16	1.39 ± 0.3
	*n* = 4	*n* = 7	*n* = 5				
All countries	84.44 ± 15.5	68.30 ± 21.3	22.90 ± 18.5		1−5	63	1.43 ± 0.3
	*n* = 19	*n* = 24	*n* = 20				

Note

Number of landscape circles mapped corresponds to the number of vineyards sampled per country. Mean ± *SD* Shannon Landscape Diversity Index (SHDI) per country.

Floral resource availability was visually estimated at every sampling date and along every inter‐row by the flower coverage of all entomophilous plants in five categories (<1% = very low; 1%–5% = low; 5%–25% = medium; 25%–50% = high; and 50%–100% = very high) following an adapted DAFOUR scale (Gardener, 2012).

### 2.2 Wild bee sampling and functional traits

Wild bees were sampled by a semiquantitative transect method in the vineyard inter‐rows. The transects length ranged between 67 and 133 m in order to adjust to the different width of the inter‐rows (1.5–3 m). To consider temporary vegetation cover management, each transect included two neighboring inter‐rows. Each vineyard was sampled five times in 2016 for 15 min per sampling event. Sampling dates among the countries were synchronized to grapevine phenology (first budburst, first flower buds, full florescence, pea‐sized berries, and beginning of maturation) to adapt to the different climatic zones (Bauer, Regner, & Schildberger, 2013). During the sampling process, each transect was walked slowly and wild bees were collected with an aerial net and later identified in the laboratory.

Functional traits of wild bees (Table 2) were selected according to the possible response to management, floral resource availability, and/or landscape diversity. Information on functional traits was gathered from the literature (Scheuchl & Willner, 2016) or expert's evaluation. As a proxy for the activity range and body size, we measured the intertegular distance (ITD in mm) with a digital microscope (Keyence VHX‐5000) of 1–5 specimens from each species and averaged per species. This shortest linear distance between the bee's wings at the dorsal side of the thorax corresponds to the size of wing muscles and to the activity range of a species (Greenleaf, Williams, Winfree, & Kremen, 2007).

**Table 2:** Wild bee functional traits used as response variables in this study

Trait	Variable type	Definition	Rationale for selection
Nesting type	Ground nesting	Majority of wild bee species in Europe excavate nest in the ground	Interlinked with habitat requirements (e.g., bare compact ground or pre‐existing cavities) which alter bee diversity and abundance
	Above‐ground nesting	Nesting in pre‐existing cavities, plant stems, dead wood (incl. *Bombus*spp.)	
	Parasitic	♀ lay their eggs in nests of specific host species	Less efficient pollinators (Garibaldi et al., 2015) but indicates vital host populations (Hudson, Dobson, & Lafferty, 2006)
Sociality	Solitary	Nest establishment and resource collection by each ♀ alone	Type of sociality could result in shorter (solitary) or longer seasonal activity (eusocial) and may affect duration in which a species is pollinating. Affected by vegetation management due to nesting type.
	Eusocial	Division of tasks: egg‐laying ♀ and ♀ that collect resources (e.g., bumble bees, some Halictidae species)	
	Parasitic	See above	See above
Body size	ITD (mm)	The shortest linear distance measured between a wing tegulae across the dorsal thorax (Cane, 1987)	Strongly related to the flying distance of a species (i.e., the distance a female can fly to collect pollen and nectar; and affected by landscape features (Gathmann & Tscharntke, 2002; Greenleaf et al., 2007; Zurbuchen et al., 2010)
Lecty	Polylectic	Pollen generalists: Pollen is collected on different plant taxa but species can show a certain degree of flower constancy	A greater variety of plants is visited to collect pollen and nectar
	Oligolectic	Pollen specialists: Pollen is collected from closely related or single plant taxa	Morphological adaption to respective flower structure; occurrence of host plant is relevant

Note

Sociality was defined as by Michener (2007).

### 2.3 Landscape survey

A 750 m radius around each sampled vineyard center was chosen for the landscape survey to get a minimum distance of 1,500 m between the study sites which covers the foraging distance of many wild bee species (Zurbuchen et al., 2010; Zurbuchen & Müller, 2012). In each landscape circle, the landscape structures following the EUNIS habitat classification (European Environment Agency (EEA), 2016) were mapped in the field during July 2015 (Austria) and between April and October 2016 (Spain, France, Romania). If available, country‐specific data sets were used as baselines (Austria: BMLFUW, 2012; Spain: Consejería de Agricultura Pesca y Desarrollo Rural, 2011; France: IGN Institut Géographique National, 2012). Digitalization and conversions to raster data were done in ArcGIS 10.2 (ESRI, 2013). The SHDI (Shannon Landscape Diversity Index) of each landscape circle was calculated in FRAGSTATS v4.2 (McGarigal, Cushman, & Ene, 2012).

### 2.4 Data analysis

Honey bee (*Apis mellifera*) counts were excluded from the main analysis, because their abundance to a great extent depends on the location of nearby beekeepers' hives (cf. Carrié et al., 2017). However, considering the pollination services honey bees provide, their abundance was compared between the different management intensities. All statistical analyses were computed in R 3.4.3 (R Core Development Team, 2018). Collinearity among predictors was assessed by scatterplots and by testing significant correlations with Spearman correlation tests (significance level = *α* ≤ 0.05).

The response variables species richness and abundance were aggregated across all sampling dates per vineyard. The predictor variables vegetation cover (proxy for vegetation management intensity) and floral resource availability were averaged per vineyard. Floral resource availability was represented by three classes (“very low,” “low,” and “medium”) after averaging, due to missing observations of the levels “high” and “very high.” The SHDI was used as index for landscape diversity because it was least collinear with the other predictors and therefore the best option to model its interactions with management intensity and floral resource availability.

Wild bee traits were summarized by community weighted means (CWM; R package “FD” Laliberté, Legendre, & Shipley, 2015). To evaluate significantly associated wild bee traits in vineyards, a PCA was constructed, including a Hellinger transformation to correct for the “arch effect” (Zuur, Ieno, & Smith, 2007). Further, the CWMs were fitted onto the PCA by vector fitting (with the “envfit” function of the “vegan” package; Oksanen et al., 2017). This function calculates the correlation and associated *p*‐values (*α* ≤ 0.05) between the ordination of species assemblage per plot and the explanatory variables by random permutations (*n* = 999; Oksanen, 2015). Finally, generalized linear models (GLMs) were used to analyze the effects of the three predictors on these significant associated traits (i.e., sociality and body size). As response variables, we used the CWMs of the body size and for sociality the number of eusocial and solitary species and their abundances.

Model selection was based on an information theoretic approach (Burnham & Anderson, 2002), and a candidate model set of 10 GLMs was formulated with different combinations of vegetation cover, floral resource availability, and SHDI and their interactions (Table 3). The country was used as predictor in every model to encompass country‐specific effects. Species richness and abundance models were formulated as GLMs with Poisson and ITD as GLMs with Gaussian error distribution. Models were ranked by the second‐order Akaike's information criterion (AICc; R package “AICcmodavg” Mazerolle, 2016). The cutoff rate to decide whether a model was the most parsimonious compared to the others was set at ΔAICc ≥ 2 (Motulsky & Christopoulos, 2003). Plots of relevant effects of the most parsimonious models were computed with the R package “effects” (Fox, 2003).

**Table 3:** Candidate models and background hypothesis according to research questions

Background hypothesis	Candidate models
Intercept‐only model	*x* ~ 1
Exclusive effect of countries	*x* ~ Country
Effect of single predictors and countries	*x* ~ Floral resource availability +Country
	*x* ~ Vegetation coverage +Country
	*x* ~ SHDI +Country
Effect of single predictors and interaction with country	*x* ~ Floral resources availability: Country
	*x* ~ Vegetation coverage: Country
	*x* ~ SHDI: Country
Extensive soil management compensates low floral resource availability in vineyards	*x* ~ Floral resource availability: Vegetation coverage +Country
Combined effects of floral resource availability, vegetation management and landscape diversity	*x* ~ Floral resources availability +Vegetation coverage +SHDI + Country
Increased landscape diversity compensates low floral resource availability or intensive management	*x* ~ Floral resources availability * SHDI +Country
	*x* ~ Vegetation coverage * SHDI +Country

Note

SHDI: Shannon Diversity Landscape Index;* x*:Response variables (wild bee species richness: total, eusocial, solitary; wild bee abundance: total, eusocial, solitary; community weighted mean (CWM) of body size.

Model quality was assessed by diagnostic plots, dispersion values, and explained deviance ($R_{\text{GLM}}^{2}$). The model quality of eusocial wild bee GLMs appeared to be distorted because only one eusocial species (three individuals) was observed in Spain. Therefore, the Spanish vineyards were excluded from models with eusocial response variables. The most parsimonious model did not change noteworthy, but model quality improved.

## 3 RESULTS

In total, 113 species and 719 individuals were sampled in vineyards across Europe (species list: Supporting Information Appendix S1: Table S1) and 217 honey bee individuals were counted. Austrian vineyards represented the highest wild bee diversity (64 species) followed by Romania (38 species), France (35 species), and Spain (20 species). Accordingly, the highest wild bee abundance was found in Austrian vineyards (329 individuals), followed by France (181 individuals), Spain (134 individuals), and Romania (77 individuals). Honey bees were most abundant in Austria (128 individuals), followed by Romania (59 individuals), France (23 individuals), and Spain (7 individuals). Honey bee abundance was significantly influenced by management intensity (Kruskal–Wallis test: *χ* ^2 ^= 9.61; *df *= 2; *p* = 0.01) being highest in temporary vegetated inter‐rows (on average 4.92 ± 6.95 individuals ± *SD*) and lowest in bare soil vineyards (1.25 ± 2.73 individuals). Regarding wild bees, *Lasioglossum marginatum* (most abundant species in Austria) and *L. malachurum* (most abundant species in France) represented together 23.4% of all sampled wild bee individuals. In Spain, *Andrena tenuistriata* was most abundant (49.2%), and in Romania, *Halictus simplex* encompassed 14.3% of the individuals. In total, 46 species were represented by only one individual. On average, the highest species numbers were sampled during the period when the first flower buds appeared (1.49 ± 1.94; Supporting Information Appendix S1: Figure S1a) and during full florescence (1.46 ± 1.94; Supporting Information Appendix S1: Figure S1a) of the vines. The highest mean (± *SD*) abundances of wild bees (2.84 ± 4.61; Supporting Information Appendix S1: Figure S1b) were also sampled when the first flower buds appeared on the vines. The lowest mean species richness and abundance were sampled at the last sampling date when the grapes started to mature (Supporting Information AppendixS1 Figure S1).

**Figure 2:**
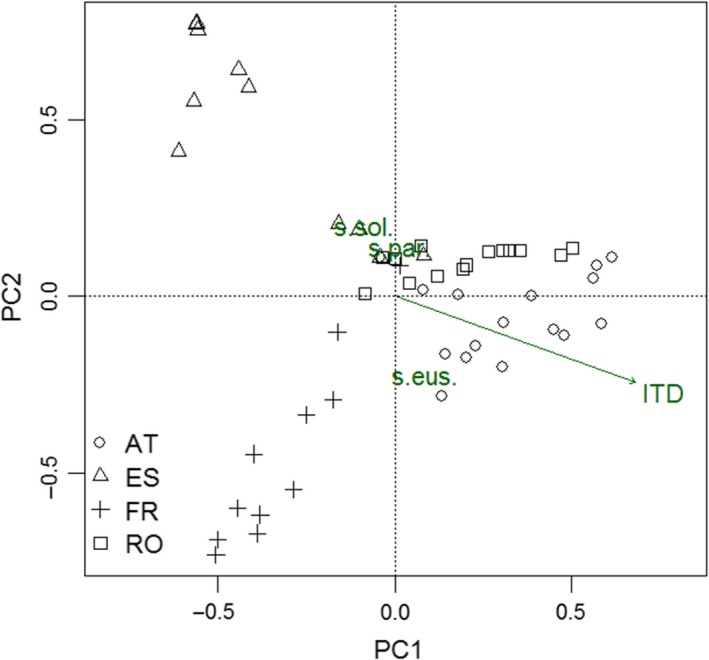
PCA for wild bee species assemblage in vineyards across Europe including wild bee traits based on significantly (*p* ≤ 0.05) correlated CWM (community weighted means) values derived by vector fitting with permutation tests (*n* = 999). ITD = Intertegular distance; s.sol = solitary wild bee species; s.par = parasitic wild bee species; s.eus = eusocial wild bee species

Overall, 65% of all wild bee species were ground nesting and 25% were above‐ground nesting. The majority (76%) of wild bee species in vineyards were polylectic and only 14% were oligolectic. Further, wild bee fauna of vineyards consisted of 26% eusocial species, 60% solitary species, and 4% species with insufficient information on sociality. Parasitic wild bees were dominant in three vineyards (two temporary and one permanently vegetated) and represented 10% of all species. The CWM of body size ranged from 0.9 to 3.0 mm ITD and was significantly related to the Austrian wild bee assemblages. Indeed, the mean (±*SD*) CWM of ITD was highest in Austria (2.10 ± 0.47 mm), followed by Romania (1.84 ± 0.44) and France (1.83 ± 0.52), and was lowest in Spanish vineyards (1.61 ± 0.53 mm). The fitted CWM revealed that sociality (*p* = 0.001) and body size (*p* = 0.01) were significantly parameters of the PCA (Figure 2).

The PCA revealed that the wild bee communities were clustered by country. Vineyards in Austria and Romania represented more similar species assemblages compared to Spain and France with more divergent wild bee communities. Further, eusocial wild bee species were characteristic for Austrian and French vineyards based on the high abundance of *Lasioglossum marginatum* in Austria and *Lasioglossum malachurum* in France.

In general, wild bee diversity, abundance and the functional traits that were significantly associated with the PCA's ordination in vineyards, were best explained by models including both floral resource availability and vegetation cover and their interaction (Table 4, Supporting Information Appendix S1: Table S2: GLM results). The average floral resource availability was generally low, but highest in Austrian and Spanish inter‐rows and lowest in Romanian inter‐rows (Supporting Information Appendix S1: Figure S1).

**Table 4:** Model selection according to AICc for each response variable

Models	Wild bee species richness			Wild bee abundance			CWM
	Total	Eusocial	Solitary	Total	Eusocial	Solitary	ITD
*x* ~ 1	471.24	239.09	306.40	1,052.9	661.58	622.47	**82.61**
*x* ~ Country	370.02	210.33	277.83	868.83	520.67	555.91	**82.38**
*x* ~ Floral resource av. + Country	303.50	170.62	248.34	607.33	309.74	485.27	85.87
*x* ~ Vegetation cov. + Country	316.79	183.68	253.21	679.20	412.68	478.09	84.50
*x* ~ SHDI +Country	371.02	212.61	277.70	866.10	522.17	551.13	**83.85**
*x* ~ Floral resource av.:Country	308.29	178.13	255.19	604.51	308.88	488.84	95.64
*x* ~ Vegetation cov.:Country	317.27	182.65	253.53	676.80	408.05	484.16	85.42
*x* ~ SHDI:Country	371.19	212.6	278.08	867.89	527.86	546.78	**82.88**
*x* ~ Floral resource av.:Vegetation cov. + Country	290.12	**169.45**	**243.39**	**535.55**	299.74	**434.27**	88.83
*x* ~ Floral resource av. + Vegetation cov. + SHDI +Country	**287.01**	166.35	**241.37**	548.32	299.15	443.55	89.73
*x* ~ Floral resource av. * SHDI +Country	310.12	176.61	255.50	613.74	**293.28**	477.73	92.27
*x* ~ Vegetation cov. * SHDI +Country	321.37	187.89	256.60	679.35	412.66	473.60	88.66

Note

AICc of the most parsimonious models for each response in bold.

CWM: Community weighted mean; ITD: Intertegular distance; *x*:Response variable; SHDI: Shannon Diversity Landscape Index

**Figure 3:**
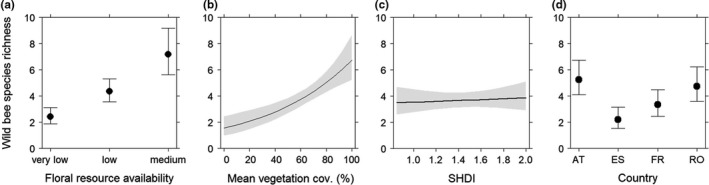
Wild bee species richness in vineyard inter‐rows in four different countries in response to (a) floral resource availability, (b) vegetation cover (%), (c) landscape diversity (SHDI: Shannon Landscape Diversity Index), and (d) countries. Error bars/gray shading: 0.95 confidence intervals

The total wild bee species richness in vineyards increased with higher floral resource availability (Figure 3a) and vegetation cover (Figure 3b), whereas landscape diversity had only a minor positive effect (Figure 3c). The significant effect of the countries on wild bee species richness in the inter‐rows (Figure 3d) is reflected in the species numbers reported from each county.

**Figure 4:**
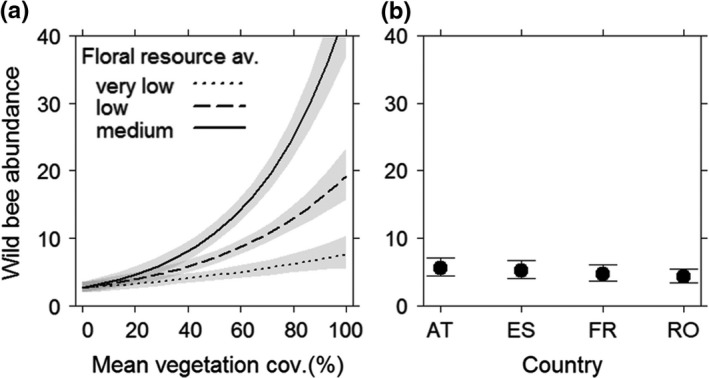
Wild bee abundance in vineyard inter‐rows in response to (a) the interaction of vegetation cover (%) and floral resource availability, and (b) countries. Error bars/gray shading: 0.95 confidence intervals

Total wild bee abundance increased by significant interactions of higher floral resource availability and mean vegetation cover. Thus, extensive vegetation management increased wild bee abundance even if floral resources were low or very low. Maximum values could be observed when floral resource availability was medium and vegetation cover greater than 60% (Figure 4a). The country effect improved the model fit but had a negligible effect on wild bee abundance (Figure 4b).

**Figure 5:**
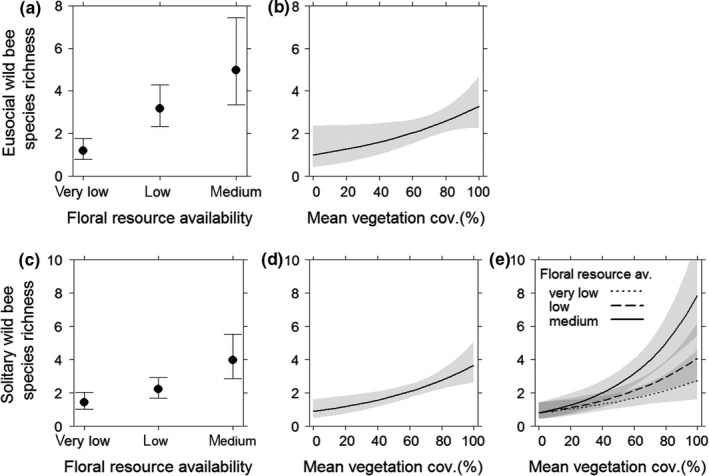
Eusocial wild bee species richness in response to (a) floral resource availability and (b) vegetation cover and solitary wild bee species richness in response to (c) floral resource availability, (d) vegetation cover, and (e) the interaction between floral resource and vegetation cover. Error bars/gray shading: 0.95 confidence intervals

**Figure 6:**
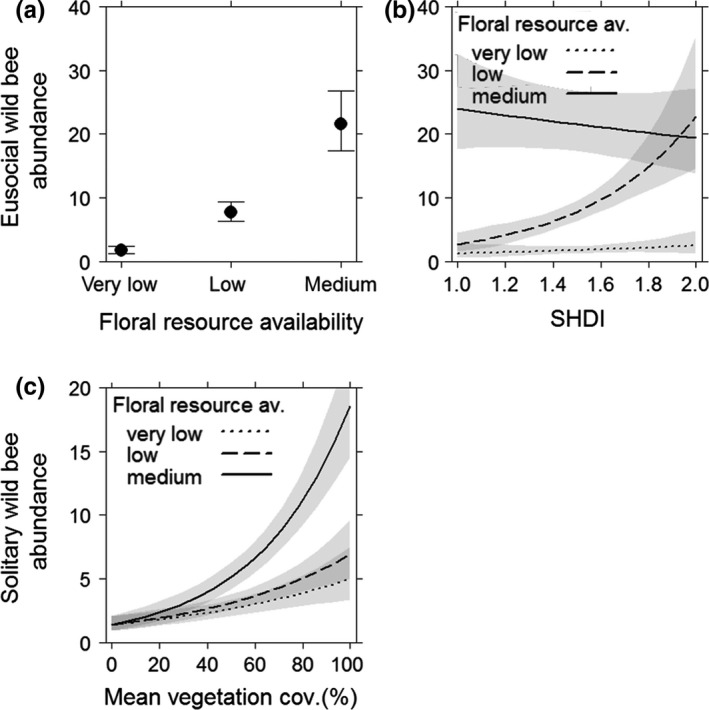
Eusocial wild bee abundance in response to (a) floral resource availability and (b) the interaction of landscape diversity and floral resource availability. Solitary wild bee abundance in response to (c) interacting effects of floral resource availability and vegetation cover. Error bars/gray shading: 0.95 confidence intervals

Eusocial as well as solitary wild bee species richness was significantly higher by increasing floral resource availability (Figure 5a,c) and mean vegetation cover (Figure 5b,d). Eusocial wild bee abundance also increased with higher floral resources (Figure 6a). Further, high landscape diversity compensated for low floral resource availability in vineyard inter‐rows and led to increased eusocial wild bee abundance. Medium floral resources in vineyard inter‐rows enhanced eusocial wild bee abundance even in simple landscapes (Figure 6b). Extensive vegetation management strategies increased solitary wild bee diversity (Figure 5e) and abundance (Figure 6c) even if low or very low floral resources were available in the inter‐rows, while higher floral resources partly compensated for the negative effect of intensive vegetation management.

Except for the significant interaction between landscape diversity and floral resource availability on eusocial wild bee abundance, landscape diversity played a secondary role for eusocial and solitary wild bee species richness and abundance (Supporting Information Appendix S1: Table S2).

Wild bee body size was equally well explained by models that included the country, the landscape diversity, or the interaction of both. However, the intercept‐only model was ranked within the most parsimonious models (Table 4) and the explained deviance of the mentioned models was low ($R_{\text{GLM}}^{2}$ = 12%–16%; Supporting Information Appendix S1: Table S1) which implies the low explanatory value of the chosen predictors for wild bee body size in vineyards.

## 4 DISCUSSION

Wild bee species richness, abundance, and functional traits in vineyard inter‐rows strongly increased with higher floral resource availability and extensive inter‐row vegetation management. Further, the total wild bee abundance as well as the diversity and abundance of solitary wild bees were significantly positively affected by the interaction of higher floral resources in extensively managed vineyard inter‐rows. The surrounding landscape had a limited influence on wild bee species richness, abundance, and most functional traits. However, it played an important role for eusocial wild bees in compensating for low floral resource availability. Most of the wild bee species and individuals were ground nesting, solitary, and generalists regarding the plants they forage on.

Across the studied vineyards, 5.7% of the almost 2000 European wild species (Nieto et al., 2015) were recorded. The recorded species numbers per country (between 20 and 64) corresponds to other vineyard studies. For example, 25–31 wild bee species were reported in 12 and 10 vineyards, respectively, in South Africa (Kehinde & Samways, 2012, 2014a, 2014b) and 17 species from 10 vineyards in California (Wilson et al., 2018). On average, the bee abundance (wild and honey bees) per vineyard in our study was lower compared to the South African vineyards (Europe: 15 individuals/vineyard vs. South Africa: 160 individuals/vineyard; Kehinde & Samways, 2012) as well as the Californian vineyards (96 individuals/vineyard; Wilson et al., 2018). However, different sampling methods could also be a reason for the different abundances of the studies. The effect of the country on wild bee species richness (Figure 3d) was also reflected in the clustering of the wild bee communities in vineyards according to the countries (Figure 2, Supporting Information Appendix S1). The divergent climatic, geographic, and/or floral zones of the studied countries are possible reasons for the different species assemblages (Gusenleitner, Schwarz, & Mazzucco, 2012; Nieto et al., 2015; Ortiz‐Sánchez, 2011; Polaszek & Mitroiu, 2013; Tomozei, 2010). It is notable that Spanish vineyards exhibited an unexpected low species richness even though the region in southern Spain is one of the diversity hot spots for wild bees in Europe (Nieto et al., 2015). The overall intensive inter‐row management in vineyards and the low landscape diversity in our Spanish study region are the most likely reasons for the low bee diversity. The most abundant species in Spanish vineyards, *Andrena tenuistriata*, prefers Mediterranean‐type shrublands as well as arable land as habitat (Roberts, 2014). The majority of those individuals (64.6%) were present in vineyards with temporary vegetation cover which demonstrates the benefit of less intensive disturbance for this ground‐nesting species. Austrian vineyards comprised the highest wild bee diversity which conforms with the generally high wild bee diversity in eastern Austria (Nieto et al., 2015). Further, the landscape diversity was highest in the Austrian wine‐growing region and inter‐row vegetation treatments included the two least intensive managements.

The strong positive effect of increased floral resources on wild bees found in this study was already documented in other agroecosystems (Scheper et al., 2015; Westphal, Steffan‐Dewenter, & Tscharntke, 2009), vineyards in South Africa (Kehinde & Samways, 2014a, 2014b) and California (Wilson et al., 2018), and natural or seminatural habitats (Haaland et al., 2011; Rollin et al., 2013). Furthermore, other pollinators like butterflies also respond positively to suitable nectar resources and larval host plants in wine‐growing areas (Gillespie & Wratten, 2012).

Vineyard inter‐rows are linear landscape elements and are comparable with flowering strips or field margins which can improve pollinator diversity, abundance, and pollination services for insect‐pollinated crops (Haaland et al., 2011; Williams et al., 2015). The positive effect of increased floral resource availability in vineyards has to be examined critically because the attraction of wild bees could lead to increased pesticide exposure of these pollinating insects. However, the effect of pesticides and their active ingredients, which are used in viticulture, on wild bee diversity, abundance, and traits, was not studied and should be addressed in future research.

The strong positive effect of extensive vegetation management agrees with other studies reporting the benefits of extensive agricultural management practices for wild bees in different crop systems (Nicholson et al., 2017; Shuler, Roulston, & Farris, 2005), as well as vineyards (Kehinde & Samways, 2012, 2014a, 2014b). Moreover, a recent meta‐analysis confirmed that positive affect of extensive management on overall biodiversity and ecosystem services (Winter et al., 2018). Ground‐nesting bees benefit from undisturbed soil conditions and can utilize vineyard inter‐rows as nesting habitat. Indeed, during field work, nesting activity of *Lasioglossum marginatum* and *L. lineare* was occasionally observed. In total, most eusocial (70%) and solitary (70%) species were ground nesting, but nesting types were not significantly associated with the PCA and not analyzed further with GLMs. In general, the high proportion of ground‐nesting wild bees is characteristic for agroecosystems because nesting habitats are widely available (e.g., unsealed roads, field verges, bare ground below vine rows). Whereas structures for above‐ground nesting wild bees (e.g., old plant material, deadwood elements) are often less abundant (Zurbuchen & Müller, 2012).

Further, floral resources are destroyed by frequent soil tillage or herbicide use in bare soil vineyards which amplifies the negative effect of intensive vegetation management. The combined positive effect of higher floral resource availability and vegetation cover on the total wild bee abundance is associated with the high abundance (79%) of ground‐nesting eusocial wild bees. The remaining 21% eusocial (above‐ground nesting) individuals were represented by bumblebees. These species colonize pre‐existing cavities below, on or above, the ground for nesting and are much likely to be negatively affected by frequent soil disturbance. The same combined positive effects on solitary wild bees are explained by the high abundance (86%) and species richness (72%) of ground‐nesting solitary wild bee species.

Even though we found a positive effect of landscape diversity on wild bee species richness, it was low, which could be explained by the superior effect of floral resource availability in the inter‐rows. These results disagree with other studies which revealed the essential importance of landscape structures on wild bee communities (Kennedy et al., 2013; Nicholson et al., 2017). Conversely, it demonstrates the necessity for increasing floral resource availability on the landscape scale to increase and maintain wild bee species richness and thus adequate pollination services for insect‐pollinated wild plants and crops (Winfree et al., 2018).

Eusocial wild bees were significantly associated with countries (Austria and Romania) where extensive inter‐row vegetation management was realized because eusocial species are more susceptible to disturbances than solitary species (Williams et al., 2010). Only eusocial wild bee abundance was affected by the interaction of SHDI and floral resource availability which could be explained by their higher vulnerability to habitat fragmentation (Williams et al., 2010). Continuous floral resource availability during the vegetation period plays a crucial role for the sexual reproduction of eusocial wild bees because a lack of pollen and nectar can lead to a colony collapse in the reproduction phase during summer (Westphal et al., 2009). Landscape structures like fallows (Toivonen, Herzon, & Kuussaari, 2016), hedges (Morandin & Kremen, 2013), solitary trees, or edges of woods (Nicholson et al., 2017; Rollin et al., 2013) provide different foraging sites for wild bees. Furthermore, these structures may compensate for negative effects of low to very low floral resource availability on eusocial wild bees that nest in the inter‐row space of vineyards (Kratschmer et al., 2018). Spanish vineyards possessed similar average floral resource availabilities as Austrian vineyards, which, according to our results, should benefit eusocial species. However, only one eusocial species was documented in Spanish vineyards. The more intensive vegetation management and low landscape diversity limited eusocial wild bee occurrence. This might decrease pollination provision at the landscape scale because pollination performance mainly depends on wild bee species richness (Winfree et al., 2018) and abundance (Winfree, Fox, Williams, Reilly, & Cariveau, 2015). Even though vines and olives, representing the dominant crops in the Spanish study region, do not rely on insect‐pollination, but other insect‐pollinated wild plants require pollination to guarantee long‐term survival. This was reported from central Europe, by Biesmeijer et al. (2006) who showed a parallel decline of wild plants and their pollinators due to insufficient pollination.

We expected that increasing average body size of bee assemblages is related to decreasing landscape diversity, because larger species can forage at greater distances (Greenleaf et al., 2007). Further, if pollen availability is low it leads to a change in maternal resource allocation to offspring, resulting in smaller adults (Renauld, Hutchinson, Loeb, Poveda, & Connelly, 2016). These effects were not observed since body size was not noteworthy affected by SHDI or by any other predictor. This is likely due to an overlapping effect by the distinct species assemblage in each country: The body size of wild bees was related to the Austrian wild bee assemblages. We explain this by the high abundance and species richness of bumble bees in Austrian vineyards compared to France, Romania, and Spain. On the other hand, a high proportion of the individuals in Spain was represented by two small wild bee species (*Andrena tenuistriata*, average 1.29 mm ITD and *Panurginus albopilosus*, average 0.89 mm ITD).

In conclusion, the total wild bee diversity and abundance as well as solitary wild bee diversity and abundance benefitted from the combination of increased floral resource availability and extensive vegetation management intensity in vineyard inter‐rows. Consequently, vineyard inter‐rows can be important habitats for wild bees in viticultural landscapes. High landscape diversity played an important role in compensating for low floral resources for eusocial wild bees. Therefore, we recommend less intensive vegetation management such as infrequent vegetation disturbance to be implemented in vineyard inter‐rows in order to achieve resilient pollination provision for insect‐pollinated crops and wild plants in viticultural landscapes. Beside enhancing wild bee diversity and abundance through these measures also honey bees will benefit which is especially important for the pollination of mass flowering crops (Brittain, Williams, et al., 2013). The implementation of pollinator‐friendly management ultimately benefits other ecosystem services like for example soil erosion mitigation, surface water runoff reduction, or biological pest control as well as biodiversity conservation (Wratten et al., 2012). Many of those ecosystem services are relevant for winegrowers and positively affected by extensive inter‐row management intensities in vineyards (Winter et al., 2018). For example, extensive vegetation management significantly improves soil loss mitigation (Winter et al., 2018), which is highly relevant in vineyards that are situated on hilly terrain. Extensive management contributes to sustainable farming contributing to the UN sustainable development goals responsible consumption and production as well as life on land (UN, 2015). Further, biodiversity‐friendly vineyard management practices (e.g., organic farming) are increasingly demanded by consumers (Schütte & Bergmann, 2019).

## CONFLICT OF INTERESTS

The authors declare no competing financial interests.

## AUTHOR CONTRIBUTION

SK, BP, SW, JGZ, AN, FB, and MG conceived and planned the experiment; SK, DP, AF, and LM conducted the field work; SK and BP identified wild bee species; SK, MS, JAE, AF, AH, and MG did landscape mapping in the field, digitalization and GIS work; SK, SW, and DP analyzed the data; all other authors were involved in significant parts of the study, wrote and/or reviewed the manuscript.

## Data Availability

Data available via Zenodo under: https://doi.org/10.5281/zenodo.2567423.
