# Supplementary material for: Response of wild bee diversity, abundance, and functional traits to vineyard inter‐row management intensity and landscape diversity across Europe
Source: Ecol Evol. 2019 Mar 12;9(7):4103–15. doi: 10.1002/ece3.5039 (PMC6467850; doi:10.1002/ece3.5039)
Supplement: Supplementary file 1 [file ECE3-9-4103-s001.docx]

**Appendix S1**

**Table S1**: Species list of wild bees from vineyards across Europe including information on total abundance, abundance per country and functional traits. Nomenclature after Fauna Europaea (Polaszek and Mitroiu 2013); *A. danuvia* (Stöckhert and Pittioni 1950), *Hylaeus dilatatus* (Notton and Dathe 2008) and *H. intermedius* (Dathe et al. 2016)

| Traits  Taxa | Nesting type | Sociality | Lecty | ITD (mm) | Abundance | | | | |
| --- | --- | --- | --- | --- | --- | --- | --- | --- | --- |
|  |  |  |  |  | Σ | AT | ES | FR | RO |
| *Andrena aerinifrons* Dours, 1873 | G | solitary | pl | 1.59 | 6 | 0 | 6 | 0 | 0 |
| *Andrena combaella* Warncke, 1966 | G | solitary | ol | 2.34 | 1 | 0 | 0 | 0 | 1 |
| *Andrena dorsata* (Kirby, 1802) | G | solitary | pl | 2.04 | 1 | 1 | 0 | 0 | 0 |
| *Andrena ferrugineicrus* Dours, 1872 | G | solitary | pl | 2.18 | 2 | 0 | 2 | 0 | 0 |
| *Andrena flavipes* Panzer 1799 | G | solitary | pl | 2.08 | 17 | 8 | 2 | 1 | 6 |
| *Andrena florentina* Magretti, 1883 | G | solitary | pl | 2.41 | 3 | 0 | 3 | 0 | 0 |
| *Andrena gravida* Imhoff, 1832 | G | solitary | pl | 2.82 | 5 | 5 | 0 | 0 | 0 |
| *Andrena labialis* (Kirby, 1802) | G | solitary | ol | 2.32 | 2 | 2 | 0 | 0 | 0 |
| *Andrena longibarbis* Perez, 1895 | G | solitary | pl | 1.59 | 1 | 0 | 1 | 0 | 0 |
| *Andrena nitida* (Müller, 1776) | G | solitary | pl | 3.02 | 1 | 0 | 1 | 0 | 0 |
| *Andrena ovatula* (Kirby, 1802) | G | solitary | pl | 2.07 | 22 | 20 | 0 | 1 | 1 |
| *Andrena pusilla* Perez, 1903 | G | solitary | pl | 1.10 | 2 | 0 | 1 | 0 | 1 |
| *Andrena saxonica* Stoeckhert, 1935 | G | solitary | ol | 1.36 | 1 | 0 | 0 | 0 | 1 |
| *Andrena simontornyella* Noskiewicz, 1939 | G | solitary | pl | 1.30 | 2 | 2 | 0 | 0 | 0 |
| *Andrena subopaca* Nylander, 1848 | G | solitary | pl | 1.42 | 2 | 0 | 0 | 0 | 2 |
| *Andrena tenuistriata* (Perez, 1895) | G | solitary | pl | 1.29 | 65 | 0 | 65 | 0 | 0 |
| *Andrena varia* Perez, 1895 ☼ | G | solitary | pl | 2.10 | 6 | 0 | 6 | 0 | 0 |
| *Anthidium manicatum* (Linnaeus, 1758) | A | solitary | pl | 3.19 | 1 | 0 | 0 | 0 | 1 |
| *Anthophora crinipes* Smith, 1854 | G | solitary | pl | 3.72 | 1 | 0 | 0 | 0 | 1 |
| *Anthophora plumipes* (Pallas, 1772) | G | solitary | pl | 3.74 | 1 | 1 | 0 | 0 | 0 |
| *Apis mellifera* (Linnaeus, 1758) | A | social | pl | n.a. | 217 | 128 | 7 | 23 | 59 |
| *Bombus haematurus* Kriechbaumer, 1870 | A | eusocial | pl | 3.65 | 1 | 1 | 0 | 0 | 0 |
| *Bombus hortorum* (Linnaeus, 1761) | A | eusocial | pl | 3.35 | 3 | 3 | 0 | 0 | 0 |
| *Bombus humilis* Illiger, 1806 | A | eusocial | pl | 3.51 | 1 | 0 | 0 | 1 | 0 |
| *Bombus lapidarius* (Linnaeus, 1758) | A | eusocial | pl | 3.65 | 33 | 30 | 0 | 3 | 0 |
| *Bombus lucorum* (Linnaeus, 1761) | A | eusocial | pl | 3.77 | 1 | 0 | 0 | 1 | 0 |
| *Bombus pascuorum* (Scopoli, 1763) | A | eusocial | pl | 3.13 | 12 | 9 | 0 | 2 | 1 |
| *Bombus pratorum* (Linnaeus, 1761) | A | eusocial | pl | 3.75 | 1 | 0 | 0 | 1 | 0 |
| *Bombus sylvarum* (Linnaeus, 1761) | A | eusocial | pl | 3.32 | 3 | 2 | 0 | 0 | 1 |
| *Bombus terrestris* (Linnaeus, 1758) | A | eusocial | pl | 3.82 | 23 | 22 | 0 | 0 | 1 |
| *Ceratina chalybea* Chevrier, 1872 | A | solitary | pl | 1.83 | 1 | 1 | 0 | 0 | 0 |
| *Ceratina cyanea* (Kirby, 1802) | A | solitary | pl | 1.26 | 3 | 1 | 0 | 2 | 0 |
| *Ceratina dalltorreana* Friese, 1896 | A | solitary | pl | 0.99 | 1 | 0 | 1 | 0 | 0 |
| *Ceratina nigrolabiata* Friese, 1896 | A | solitary | pl | 1.36 | 3 | 2 | 0 | 0 | 1 |
| *Chelostoma florisomne* (Linnaeus, 1758) | A | solitary | ol | 1.57 | 1 | 0 | 0 | 0 | 1 |
| *Eucera chrysopyga* (Perez, 1879) | G | solitary | pl | 3.21 | 2 | 1 | 0 | 0 | 1 |
| *Eucera eucnemidea* Dours, 1873 | G | solitary | pl | 2.35 | 16 | 0 | 16 | 0 | 0 |
| *Eucera interrupta* Baer, 1850 | G | solitary | ol | 2.79 | 1 | 0 | 0 | 0 | 1 |
| *Eucera longicornis* (Linnaeus, 1758) | G | solitary | ol | 3.21 | 1 | 1 | 0 | 0 | 0 |
| *Eucera nigrescens* Perez, 1879 | G | solitary | ol | 3.16 | 20 | 13 | 0 | 7 | 0 |
| *Eucera nigrilabris* Lepeletier, 1841 | G | solitary | pl | 3.65 | 2 | 0 | 2 | 0 | 0 |
| *Eucera numida* Lepeletier, 1841 | G | solitary | pl | 3.28 | 2 | 0 | 2 | 0 | 0 |
| *Eucera seminuda* Brullé, 1832 | G | solitary | pl | 2.65 | 1 | 0 | 0 | 0 | 1 |
| *Halictus eurygnathus* Bluethgen, 1931 | G | solitary | pl | 1.77 | 1 | 0 | 0 | 1 | 0 |
| *Halictus kessleri* Bramson, 1879 | G | eusocial | pl | 1.29 | 9 | 3 | 0 | 0 | 6 |
| *Halictus langobardicus* Bluethgen, 1944 | G | solitary | pl | 1.47 | 1 | 0 | 0 | 0 | 1 |
| *Halictus maculatus* Smith, 1848 | G | eusocial | pl | 1.37 | 7 | 4 | 0 | 2 | 1 |
| *Halictus quadricinctus* (Fabricius, 1776) | G | solitary | pl | 2.70 | 4 | 3 | 0 | 0 | 1 |
| *Halictus rubicundus* (Christ, 1791) | G | eusocial | pl | 2.05 | 1 | 1 | 0 | 0 | 0 |
| *Halictus scabiosae* (Rossi, 1790) | G | eusocial | pl | 2.58 | 15 | 0 | 0 | 15 | 0 |
| *Halictus seladonius* (Fabricius, 1794) | G | eusocial | pl | 1.48 | 2 | 2 | 0 | 0 | 0 |
| *Halictus simplex* Bluethgen, 1923 | G | solitary | pl | 1.77 | 26 | 13 | 0 | 2 | 11 |
| *Halictus smaragdulus* Vachal, 1895 | G | eusocial | pl | 1.22 | 2 | 1 | 0 | 0 | 1 |
| *Halictus subauratus* (Rossi, 1792) | G | eusocial | pl | 1.42 | 7 | 5 | 0 | 1 | 1 |
| *Halictus tumulorum* (Linnaeus, 1758) | G | eusocial | pl | 1.33 | 4 | 1 | 0 | 1 | 2 |
| *Heriades truncorum* (Linnaeus, 1758) | A | solitary | ol | 1.32 | 2 | 2 | 0 | 0 | 0 |
| *Hoplitis claviventris* Thomson, 1872 | A | solitary | pl | 2.01 | 1 | 1 | 0 | 0 | 0 |
| *Hoplitis leucomelana* (Kirby, 1802) | A | solitary | pl | 1.43 | 3 | 1 | 0 | 1 | 1 |
| *Hoplitis tuberculata* Nylander, 1848 | A | solitary | pl | 2.47 | 2 | 0 | 0 | 2 | 0 |
| *Hoplosmia spinulosa* (Kirby, 1802) | A | solitary | ol | 1.84 | 2 | 0 | 0 | 1 | 1 |
| *Hylaeus communis* Nylander, 1852 | A | solitary | pl | 0.96 | 1 | 1 | 0 | 0 | 0 |
| *Hylaeus dilatatus* (Kirby, 1802) | A | solitary | pl | 1.20 | 1 | 0 | 0 | 0 | 1 |
| *Hylaeus intermedius* Förster, 1871 ☼ | A | solitary | pl | 1.33 | 1 | 0 | 0 | 0 | 1 |
| *Hylaeus variegatus* (Fabricius, 1798) | A | solitary | pl | 1.37 | 1 | 0 | 0 | 1 | 0 |
| *Lasioglossum aeratum* (Kirby, 1802) | G | eusocial | pl | 0.98 | 2 | 2 | 0 | 0 | 0 |
| *Lasioglossum brevicorne* (Schenck, 1870) | G | eusocial | ol | 1.43 | 1 | 0 | 0 | 1 | 0 |
| *Lasioglossum calceatum* (Scopoli, 1763) | G | eusocial | pl | 1.74 | 5 | 4 | 0 | 0 | 1 |
| *Lasioglossum discum* (Smith, 1853) | G | solitary | pl | 2.33 | 2 | 2 | 0 | 0 | 0 |
| *Lasioglossum glabriusculum* (Morawitz, 1872) | G | eusocial | pl | 0.81 | 2 | 2 | 0 | 0 | 0 |
| *Lasioglossum griseolum* (Morawitz, 1872) | G |  | pl | 1.00 | 2 | 2 | 0 | 0 | 0 |
| *Lasioglossum interruptum* (Panzer, 1798) | G | eusocial | pl | 1.44 | 4 | 1 | 0 | 2 | 1 |
| *Lasioglossum laevigatum* (Kirby, 1802) | G | solitary | pl | 2.10 | 2 | 1 | 0 | 1 | 0 |
| *Lasioglossum laterale* (Brullé, 1832) | G |  | pl | 1.86 | 1 | 1 | 0 | 0 | 0 |
| *Lasioglossum lativentre* (Schenk, 1853) | G | solitary | pl | 1.38 | 6 | 4 | 0 | 2 | 0 |
| *Lasioglossum leucozonium* (Schrank, 1781) | G | solitary | pl | 1.72 | 3 | 1 | 0 | 2 | 0 |
| *Lasioglossum lineare* (Schenk, 1869) | G | eusocial | pl | 1.36 | 20 | 20 | 0 | 0 | 0 |
| *Lasioglossum malachurum* (Kirby, 1802) | G | eusocial | pl | 1.55 | 90 | 6 | 3 | 81 | 0 |
| *Lasioglossum marginatum* (Brullé, 1832) | G | eusocial | pl | 1.65 | 79 | 70 | 0 | 0 | 9 |
| *Lasioglossum mesosclerum* (Perez, 1903) | G |  | pl | 1.22 | 1 | 1 | 0 | 0 | 0 |
| *Lasioglossum minutissimum* (Kirby, 1802) | G | solitary | pl | 0.80 | 6 | 6 | 0 | 0 | 0 |
| *Lasioglossum morio* (Fabricius, 1793) | G | eusocial | pl | 1.02 | 9 | 0 | 0 | 5 | 4 |
| *Lasioglossum nigripes* (Lepeletier, 1841) | G | eusocial | pl | 1.94 | 1 | 1 | 0 | 0 | 0 |
| *Lasioglossum pauperatum* (Brullé, 1832) | G |  | pl | 1.08 | 2 | 0 | 1 | 1 | 0 |
| *Lasioglossum pauxillum* (Schenck, 1853) | G | eusocial | pl | 1.18 | 25 | 3 | 0 | 22 | 0 |
| *Lasioglossum punctatissimum* (Schenck, 1853) | G | solitary | pl | 1.19 | 2 | 0 | 0 | 2 | 0 |
| *Lasioglossum puncticolle* (Morawitz, 1872) | G | eusocial | pl | 1.53 | 1 | 0 | 0 | 1 | 0 |
| *Lasioglossum pygmaeum* (Schenck, 1853) | G | solitary | pl | 1.15 | 1 | 1 | 0 | 0 | 0 |
| *Lasioglossum quadrinotatum* (Kirby, 1802) | G | solitary | pl | 1.72 | 1 | 1 | 0 | 0 | 0 |
| *Lasioglossum villosulum* (Kirby, 1802) | G | solitary | pl | 1.18 | 11 | 0 | 1 | 10 | 0 |
| *Lasioglossum xanthopus* (Kirby, 1802) | G | solitary | pl | 2.21 | 3 | 3 | 0 | 0 | 0 |
| *Lasioglossum zonulum* (Smith, 1848) | G | solitary | pl | 1.87 | 6 | 0 | 0 | 1 | 5 |
| *Megachile centuncularis* (Linnaeus, 1758) | A | solitary | pl | 2.77 | 1 | 0 | 0 | 1 | 0 |
| *Melitta leporina* (Panzer, 1799) | G | solitary | ol | 2.20 | 1 | 1 | 0 | 0 | 0 |
| *Nomada agrestis* Fabricius, 1787 | P | parasitic | pl | 2.74 | 1 | 0 | 1 | 0 | 0 |
| *Nomada basalis* Herrich-Schäffer, 1839 | P | parasitic | pl | 1.86 | 1 | 0 | 0 | 0 | 1 |
| *Nomada discrepans* Schmiedeknecht, 1882 | P | parasitic | pl | 1.08 | 4 | 0 | 4 | 0 | 0 |
| *Nomada flavoguttata* (Kirby, 1802) | P | parasitic | pl | 1.07 | 1 | 1 | 0 | 0 | 0 |
| *Osmia adunca* (Panzer, 1798) | A | solitary | ol | 2.40 | 1 | 0 | 0 | 1 | 0 |
| *Osmia aurulenta* (Panzer, 1799) | A | solitary | pl | 2.70 | 4 | 1 | 0 | 0 | 3 |
| *Osmia caerulescens* (Linnaeus, 1758) | A | solitary | pl | 2.22 | 4 | 4 | 0 | 0 | 0 |
| *Panurginus albopilosus* (Lucas, 1846) | A | solitary | pl | 0.89 | 13 | 0 | 13 | 0 | 0 |
| *Panurgus calcaratus* (Scopoli, 1763) | G | eusocial | ol | 1.66 | 1 | 1 | 0 | 0 | 0 |
| *Panurgus dentipes* Latreille, 1811 | G | solitary | ol | 1.54 | 2 | 0 | 0 | 2 | 0 |
| *Rophites quinquespinosus* Spinola, 1808 | G | solitary | ol | 2.13 | 1 | 0 | 0 | 0 | 1 |
| *Sphecodes albilabris* (Fabricius, 1793) | P | parasitic | pl | 1.82 | 1 | 0 | 0 | 0 | 1 |
| *Sphecodes ephippius* (Linnaeus, 1767) | P | parasitic | pl | 1.36 | 2 | 2 | 0 | 0 | 0 |
| *Sphecodes ferruginatus* Hagens, 1882 | P | parasitic | pl | 1.59 | 1 | 1 | 0 | 0 | 0 |
| *Sphecodes gibbus* (Linnaeus, 1758) | P | parasitic | pl | 1.27 | 1 | 1 | 0 | 0 | 0 |
| *Sphecodes ruficrus* (Erichson, 1835) | P | parasitic | pl | 1.50 | 1 | 0 | 1 | 0 | 0 |
| *Sphecodes rufiventris* (Panzer, 1798) | P | parasitic | pl | 1.25 | 1 | 1 | 0 | 0 | 0 |
| *Sphecodes schenkii* Hagens, 1882 | P | parasitic | pl | 1.47 | 1 | 0 | 0 | 0 | 1 |
| *Sphecodes* sp. | P | parasitic | pl |  | 4 | 4 | 0 | 0 | 0 |
| *Systropha curvicornis* (Scopoli, 1770) | G | solitary | ol | 1.97 | 14 | 14 | 0 | 0 | 0 |
| *Systropha planidens* Giraud, 1861 | G | solitary | ol | 2.06 | 3 | 3 | 0 | 0 | 0 |
| Total abundance | | | | | 719 | 329 | 132 | 181 | 77 |
| Total species richness | | | | | 113 | 64 | 20 | 35 | 38 |

*Nesting:* *A* above-ground nesting; *G* ground nesting; *P* parasitic

*Lecty:* *pl* polylectic; *ol* oligolectic; *P* parasitic

*ITD* Inter-tegular distance from 1-5 specimen per country (averaged if >1 specimen present)

* *Bombus terrestris*: Includes field counts of *B. terrestris* and *B. lucorum* in AT

** *Halictus simplex*: Female specimen represent a species complex including *H. simplex*. *H. eurygnathus* and *H. langobardicus* because female identification features are ambiguous (Amiet et al. 2001). One male *H. langobardicus* was identified in RO and one *H. eurygnathus* was identified in FR.

☼ First record for a country

**Appendix**

**Table S2:** Effect sizes (Estimate ± SE). p-values and model quality assessment (dispersion. R²_GLM_) of the most parsimonious models for wild bee diversity. abundance and characteristic traits in vineyards across Europe. The categories “very low” floral resource availability and country “AT” were used as baseline for parameter estimation of the categorical predictor variables.

| **Response variables** | **Predictor variables** | | **Estimate ± SE** | **p-value** | **Dispersion** | **R²_GLM_** |
| --- | --- | --- | --- | --- | --- | --- |
| Species richness |  |  |  |  |  |  |
| Total ~ | Floral resources | “low” | 0.590 ± 0.165 | ≤ 0.001 | 1.292 | 69.6 % |
|  |  | “medium” | 1.096 ± 0.187 | ≤ 0.001 |  |  |
|  | Mean veg. cov. (%) |  | 0.015 ± 0.003 | ≤ 0.001 |  |  |
|  | SHDI |  | 0.089 ± 0.219 | 0.683 |  |  |
|  | Country | “ES” | -0.875 ± 0.225 | ≤ 0.001 |  |  |
|  |  | “FR” | -0.459 ± 0.148 | 0.002 |  |  |
|  |  | “RO” | -0.106 ± 0.203 | 0.601 |  |  |
| Eusocial ~ | Floral resources | “low” | 0.985 ± 0.266 | ≤ 0.001 | 0.884 | 67.8 % |
|  |  | “medium” | 1.438 ± 0.305 | ≤ 0.001 |  |  |
|  | Mean veg. cov. (%) |  | 0.013 ± 0.006 | 0.023 |  |  |
|  | SHDI |  | 0.341 ± 0.332 | 0.303 |  |  |
|  | Country | “FR” | -0.172 ± 0.203 | 0.398 |  |  |
|  |  | “RO” | -0.082 ± 0.318 | 0.796 |  |  |
| Solitary ~ | Floral resources | “low” | 0.437 ± 0.228 | 0.056 | 1.344 | 49.9 % |
|  |  | “medium” | 1.025 ± 0.257 | ≤ 0.001 |  |  |
|  | Mean veg. cov. (%) |  | 0.014 ± 0.004 | 0.001 |  |  |
|  | SHDI |  | -0.175 ± 0.311 | 0.574 |  |  |
|  | Country | “ES” | -0.423 ± 0.278 | 0.077 |  |  |
|  |  | “FR” | -0.596 ± 0.225 | 0.008 |  |  |
|  |  | “RO” | -0.054 ± 0.278 | 0.845 |  |  |
| Solitary ~ | Mean veg. cov. (%) : Floral resources | |  |  | 3.351 | 47.0 % |
|  |  | : “very low” | 0.012 ± 0.005 | 0.009 |  |  |
|  |  | : “low” | 0.016 ± 0.005 | ≤ 0.001 |  |  |
|  |  | : “medium” | 0.023 ± 0.004 | ≤ 0.001 |  |  |
|  | Country | “ES” | -0.239 ± 0.254 | 0.346 |  |  |
|  |  | “FR” | -0.655 ± 0.222 | 0.003 |  |  |
|  |  | “RO” | -0.023 ± 0.004 | 0.748 |  |  |
| Abundance |  |  |  |  |  |  |
| Total ~ | Mean veg. cov. (%) : Floral resources | |  |  | 5.481 | 63.4 % |
|  |  | : “very low” | 0.011 ± 0.002 | ≤ 0.001 |  |  |
|  |  | : “low” | 0.019 ± 0.002 | ≤ 0.001 |  |  |
|  |  | : “medium” | 0.028 ± 0.002 | ≤ 0.001 |  |  |
|  | Country | “ES” | -0.072 ± 0.120 | 0.519 |  |  |
|  |  | “FR” | -0.174 ± 0.093 | 0.067 |  |  |
|  |  | “RO” | -0.264 ± 0.153 | 0.216 |  |  |
| Eusocial ~ | Floral resources | “low” | -0.714 ± 1.271 | 0.574 | 3.351 | 73.9 % |
|  |  | “medium” | 3.831 ± 1.160 | ≤ 0.001 |  |  |
|  | SHDI |  | 0.683 ± 0.688 | 0.321 |  |  |
|  | SHDI : Floral resources | |  |  |  |  |
|  |  | : “low” | 1.457 ± 0.799 | 0.068 |  |  |
|  |  | : “medium” | -0.895 ± 0.728 | 0.219 |  |  |
| Solitary ~ | Mean veg. cov. (%) : Floral resources | |  |  | 4.744 | 44.2 % |
|  |  | : “very low” | 0.013 ± 0.003 | ≤ 0.001 |  |  |
|  |  | : “low” | 0.016 ± 0.003 | ≤ 0.001 |  |  |
|  |  | : “medium” | 0.026 ± 0.003 | ≤ 0.001 |  |  |
|  | Country | “ES” | 0.766 ± 0.155 | ≤ 0.001 |  |  |
|  |  | “FR” | -0.681 ± 0.182 | ≤ 0.001 |  |  |
|  |  | “RO” | -0.034 ± 0.213 | 0.873 |  |  |
| Body size |  |  |  |  |  |  |
| ITD (mm) ~ | Country | “ES” | -0.491 ± 0.186 | 0.011 | 0.200 | 12.6 % |
|  |  | “FR” | -0.272 ± 0.186 | 0.149 |  |  |
|  |  | “RO” | -0.254 ± 0.178 | 0.159 |  |  |
| ITD (mm) ~ | Intercept |  | 1.866 ± 0.068 | ≤ 0.001 | 0.218 | 0.0 % |
| ITD (mm) ~ | SHDI |  | 0.292 ± 0.295 | 0.327 | 0.199 | 14.3 % |
|  | Country | “ES” | -0.395 ± 0.209 | 0.066 |  |  |
|  |  | “FR” | -0.274 ± 0.186 | 0.145 |  |  |
|  |  | “RO” | -0.189 ± 0.189 | 0.323 |  |  |
| ITD (mm) ~ | SHDI : Country |  |  |  | 0.196 | 15.8 % |
|  |  | : “AT” | 0.378 ± 0.273 | 0.172 |  |  |
|  |  | : “ES” | 0.053 ± 0.351 | 0.879 |  |  |
|  |  | : “FR” | 0.206 ± 0.279 | 0.463 |  |  |
|  |  | : “RO” | 0.257 ± 0.320 | 0.426 |  |  |

*ITD* Inter-tegular distance

*SHDI* Shannon Landscape Diversity Index

*:* Interaction of parameters

*R²_GLM_* Explained deviance = How much variation (%) of the response variable is explained by the predictor variable(s)

**Appendix – Figures:**


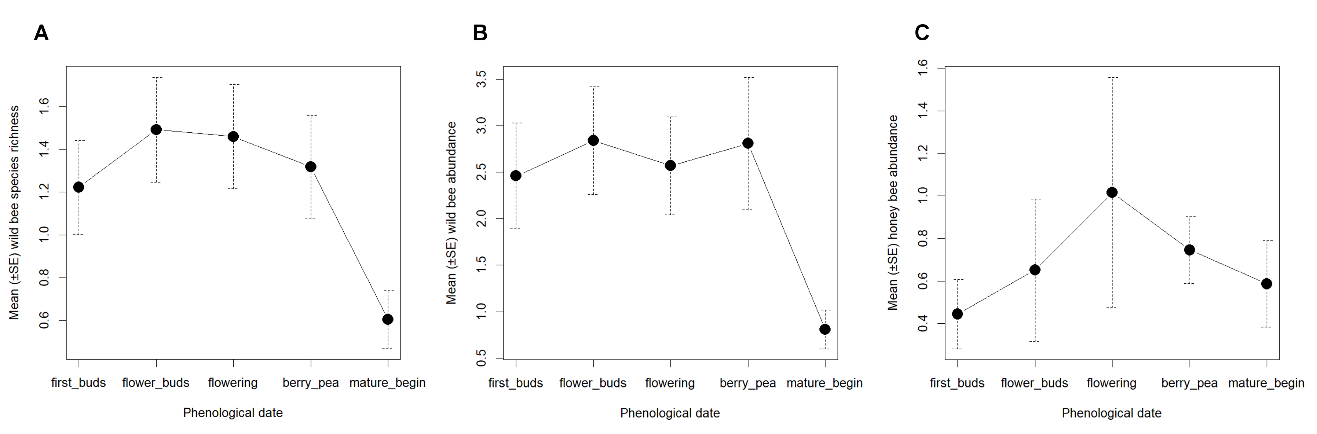


**Fig. S1.** Mean (±SE) wild bee species richness (a) abundance (b) and honey bee abundance (c) across sampling dates in vineyards that were synchronized to grapevine phenology


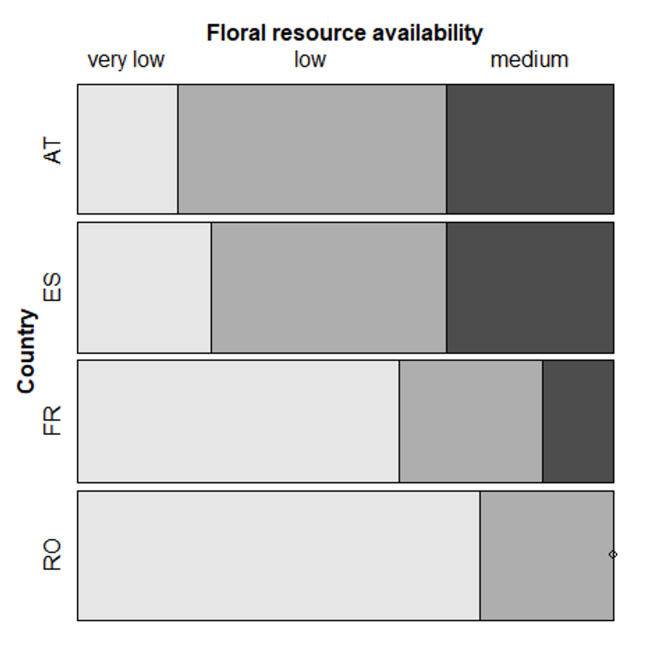


**Fig. S2.** Average floral resource availability in vineyard inter-rows in four European countries in 2016. The Mosaic plot (R package “vcd”, (Meyer et al. 2016)) visualizes the averaged (per inter-row) proportion of floral resource availability categories per country.

**Literature Appendix:**

Amiet, F., M. Herrmann, A. Müller, and R. Neumeyer. 2001. Apidae 3: Halictus, Lasioglossum. Page Fauna Helvetica 6. Schweizerische Entomologische Gesellschaft, Neuachtel.

Cane, J. H. 1987. Estimation of Bee Size Using Intertegular Span (Apoidea). Journal of the Kansas Entomological Society 60:145–147.

Dathe, H. H., E. Scheuchl, and E. Ockermüller. 2016. Illustrierte Bestimmungstabelle für die Arten der Gattung Hylaeus F. (Maskenbienen) in Deutschland, Österreich und der Schweiz. Entomologica Austriaca Supplement:51.

Garibaldi, L. A., I. Bartomeus, R. Bommarco, A. M. Klein, S. A. Cunningham, M. A. Aizen, V. Boreux, M. P. D. Garratt, L. G. Carvalheiro, C. Kremen, C. L. Morales, C. Schüepp, N. P. Chacoff, B. M. Freitas, V. Gagic, A. Holzschuh, B. K. Klatt, K. M. Krewenka, S. Krishnan, M. M. Mayfield, I. Motzke, M. Otieno, J. Petersen, S. G. Potts, T. H. Ricketts, M. Rundlöf, A. Sciligo, P. A. Sinu, I. Steffan-Dewenter, H. Taki, T. Tscharntke, C. H. Vergara, B. F. Viana, and M. Woyciechowski. 2015. Trait matching of flower visitors and crops predicts fruit set better than trait diversity. Journal of Applied Ecology 52:1436–1444.

Gathmann, A., and T. Tscharntke. 2002. Foraging ranges of solitary bees. Journal of Animal Ecology 71:757–764.

Greenleaf, S. S., N. M. Williams, R. Winfree, and C. Kremen. 2007. Bee foraging ranges and their relationship to body size. Oecologia 153:589–96.

Hudson, P. J., A. P. Dobson, and K. D. Lafferty. 2006. Is a healthy ecosystem one that is rich in parasites? Trends in Ecology and Evolution 21:381–385.

Meyer, D., A. Zeileis, and K. Hornik. 2016. vcd: Visualizing Categorical Data.

Michener, C. D. 2007. The Bees of the World. 2nd edition. The Johns Hopkins University Press, Baltimore, Maryland.

Notton, D. G., and H. H. Dathe. 2008. William Kirby’s types of Hylaeus Fabricius (Hymenoptera, Colletidae) in the collection of the Natural History Museum, London. Journal of Natural History 42:1861–1865.

Polaszek, A., and M.-D. Mitroiu. 2013. Fauna Europaea: Apidae. https://fauna-eu.org.

Stöckhert, E., and B. Pittioni. 1950. Über einige neue und verkannte Andrena-Arten (Hymenoptera, Andrenidae). Beiträge zur Kenntnis paläarktischer Apiden. III. Annalen des Naturhistorischen Museums in Wien 57:284–295.

Zurbuchen, A., L. Landert, J. Klaiber, A. Müller, S. Hein, and S. Dorn. 2010. Maximum foraging ranges in solitary bees: only few individuals have the capability to cover long foraging distances. Biological Conservation 143:669–676.
